# Mitophagy, Ferritinophagy and Ferroptosis in Retinal Pigment Epithelial Cells Under High Glucose Conditions: Implications for Diabetic Retinopathy and Age-Related Retinal Diseases

**Published:** 2021-09-27

**Authors:** Lalit Pukhrambam Singh, Thangal Yumnamcha, Takhellambam S Devi

**Affiliations:** Department of Ophthalmology, Visual and Anatomical Sciences, Wayne State University School of Medicine, USA

**Keywords:** TXNIP, Oxidative stress, Mitophagy, Ferritinophagy, Ferroptosis, RPE, Diabetic retinopathy

## Abstract

Diabetic retinopathy (DR) is a devastating disease leading to blindness among majority of working adults around the globe. Nonetheless, an effective treatment or cure for the disease is still to be achieved. This is because the cellular and molecular mechanisms of DR are complex and not fully understood yet. In this article, we describe how high glucose induced TXNIP upregulation and associated redox stress may cause mitochondrial dysfunction, mitophagy, ferritinophagy (iron release by autophagy) and lysosome destabilization. Labile irons react with hydrogen peroxide (H2O2) to generate hydroxyl radicals (.OH) by the Fenton reaction and cause membrane phospholipid peroxidation due to reduction in glutathione (GSH) level and glutathione peroxidase 4 (GPX4) activity, which cause ferroptosis, a recently identified non-apoptotic cell death mechanism. We used in this study a retinal pigment epithelial cell line, ARPE- 19 and exposed it to high glucose in *in vitro* cultures to highlight some of the intricacies of these cellular processes, which may be relevant to the pathogenesis of DR and age-related retinal neurodegenerative diseases, such as age-related macular degeneration, AMD.

## Introduction

The retina is a complex organ/tissue, a part of the central nervous system, involved in visual perception. It consumes large amounts of glucose and oxygen to generate ATP needed for its phototransduction activity via oxidative phosphorylation in the mitochondrial electron transport chain. Nonetheless, during the process of ATP production, reactive oxygen radicals are produced, which can damage mitochondrial membranes, proteins and mtDNA. Therefore, antioxidant systems are present in mitochondria to neutralize the harmful reactive oxygen species by glutathione (GSH), thioredoxin 2 (Trx_2_), superoxide dismutase 2 (SOD2) and others [1]. However, under chronic stress and diseases, such as diabetic retinopathy (DR), the reactive oxygen/nitrogen species (ROS/RNS) overwhelm the antioxidant system causing mitochondrial stress and damage. Damaged mitochondria produce less ATP but continues to generate ROS, therefore, needed to remove by a specific process of autophagy, called mitophagy [2]. To begin mitophagy, the damage part of the mitochondrion is separated by fission involving dynamin-related protein 1 (DRP-1) and mitochondrial fission protein 1 fis1. Then, the isolated damaged mitochondria are marked with ubiquitin by the Pink1-Parkin pathway [3]. Parkin is an E3 ubiquitin ligase, which adds ubiquitin to mitochondrial outer membrane proteins, including VDAC1 and Mfn2. Pink1 is an inner mitochondrial membrane protein, which is imported inside mitochondria under normal physiology and degraded by PARL, a member of the rhomboid intramembrane protease family [4]. However, when the mitochondrion is damaged or depolarized, Pink1 accumulates on the outer membrane and phosphorylates ubiquitin and other mitochondrial outer membrane proteins, which are then recognized by Parkin. Once ubiquitinated by Parkin, the damaged mitochondria bind to its adaptors, such as optineurin and p62 sequestosome 1 and the double-membrane LC3BII auto phagophore, then engulfs inside [5]. The mito-autophagosome subsequently fuses with lysosomes to form autolysosome and the contents are digested by lysosomal acidic proteases and enzymes and recycle as nutrients [6]. Mitophagy is important, as a quality control process, in fully differentiated non-dividing cells, including retinal neurons and pigment epithelial cells (RPE). These cells cannot redistribute damaged mitochondria to daughter cells. Therefore, mitophagy reduces cellular stress and inflammatory processes as damaged mitochondria release mtROS and oxidized mtDNA that are recognized by innate immune platforms, such as membrane toll-like receptors (TLRs) and cytosolic NLRP3 inflammasome [7].

To balance mitochondrial number and maintain bioenergetics, synthesis of new mitochondria is important. For this to occur, the activation of transcription factors PGC1α and TFAM are required to synthesize nuclear-encoded mitochondria-targeted genes and mitochondria genes, respectively [8]. Furthermore, several mitochondrial enzymes involved in TCA cycles, such as aconitase 2 and mitochondrial electron transport complexes (I, II and III) contain iron-sulfur complex/cluster (4Fe-4S) that is required for holoenzyme assembly and function [9]. Cellular iron is stored as ferric iron (Fe^3+^) in ferritin cages containing ferritin heavy and light chains since labile ferrous iron Fe^2+^ is redox active and reacts with hydrogen peroxide (H_2_O_2_) to generate highly reactive hydroxyl radicals (·OH), which attack on membrane lipids, proteins and DNA [10,11]. Each ferritin cage may contain up to 24 ferritin subunits (heavy and light chains) and stores up to 4500 ferric iron molecules [12]. Therefore, iron supply for mitochondrial iron-sulfur complex assembly comes from the autophagic degradation of ferritin cages in lysosomes (ferritinophagy), which releases a pool of labile Fe^2+^ in the cytosol [13,14]. These cytosolic labile irons are then transferred into the mitochondrion via mitochondrial membrane iron transporters, namely, mitoferrin 1 and 2, where they are assembled into iron-sulfur complexes into mitochondrial TCA enzymes and electron transport complexes by the iron-sulfur cluster synthesis machinery [15]. In addition, free iron (Fe^2+^) is also required for several cytosolic enzymes, including 5-lipoxygenase (ALOX-5) and nuclear DNA repair and replication enzymes as cofactors for their activity [16]. Circulating ferric iron in transferrin complex (Tf-Fe^3+^) is taken up by cell surface transferrin receptors (TfR1 and TfR2) via endocytosis. Ferric iron is then reduced to ferrous iron and releases into the cytosol by endosomal divalent metal transporter DMT1 [17]. Then, the free iron (Fe^2+^) is trapped into ferritin cages and converted to Fe^3+^ inside for storage [18]. As mentioned above, utilization of iron requires ferritinophagy, autophagic degradation of ferritin and acidic reduction of ferric to ferrous iron inside the lysosome. During chronic disease and oxidative stress, mitochondrial stress and damage occur, which are removed by mitophagy, and biosynthesis of new mitochondria require ferritinophagy. Therefore, continuous, or excess mitophagic and ferritinophagic flux to lysosomes will cause lysosomal iron accumulation, oxidative stress, increased pH, lipoprotein aggregation, lysosome enlargement and lysosomal membrane permeabilization (LMP) [19,20]. Therefore, released ferrous iron will produce reactive hydroxyl radicals, which results in membrane lipid peroxidation, including the lysosomal, mitochondrial and plasma membrane [21]. In addition, free iron also activates ALOX5, further enhancing lipid peroxidation [22]. One of the enzymes that detoxify lipid peroxidation is glutathione peroxidase 4 (GPX4), which uses 2 molecules of glutathione (GSH) [23]. However, in diabetes and under high glucose, we have shown that a protein called thioredoxin-interacting protein (TXNIP) is strongly induced in most cells examined, including retinal and renal cells [24–27]. TXNIP causes cellular oxidative stress and inflammation by binding to and inhibiting antioxidant thioredoxin (Trx) [26–28]. Trx1 is present in the cytosol and nucleus while Trx2 is the mitochondrial isoform. TXNIP is present in all cellular organelles, including mitochondria. Therefore, sustained TXNIP upregulation under high glucose environment causes cellular redox stress, mitochondrial dysfunction, and premature cell death [26,29]. Because cellular and mitochondrial stress depletes antioxidants, including GSH, the activity of GPX4 and other redox enzymes are inactivated. Cell death due to iron accumulation and lipid peroxidation due to GPX4 inactivation was recently termed as ferroptosis [30]. Mitophagy, ferritinophagy and lysosome destabilization may play critical roles in this type of ferroptotic cell death. In the present article, we propose a plausible mechanism for TXNIP and associated redox stress in mitochondrial dysfunction, mitophagy and ferritinophagy in ferroptosis in retinal RPE cells under sustained high glucose environment in culture as seen in DR. RPE cells form the outer blood-retinal-barrier (oBRB) between the neuroretina and fenestrated choriocapillaris. RPE dysfunction leads to photoreceptor death in DR and age-related retinal disease [31,32].

## Materials and Methods

The reagents, experimental procedures and assays performed in this study with human retinal pigment epithelial cell line, ARPE-19, in *in vitro* culture under high glucose (HG, 25mM) or low glucose (LG, 5.5mM) are like those described in our recent publications [33,34]. The assays include Western blots, QPCR, LDH leakage measurement, and Immunofluorescence staining. Ad-CMV-mt-Keima and Ad-CMV- LAMP1-mCherry were produced by Vector Biosystems, Inc., Malvern, USA. A summary of reagents used are shown in Table 1.

## Results and Discussion

**Figure 1:**
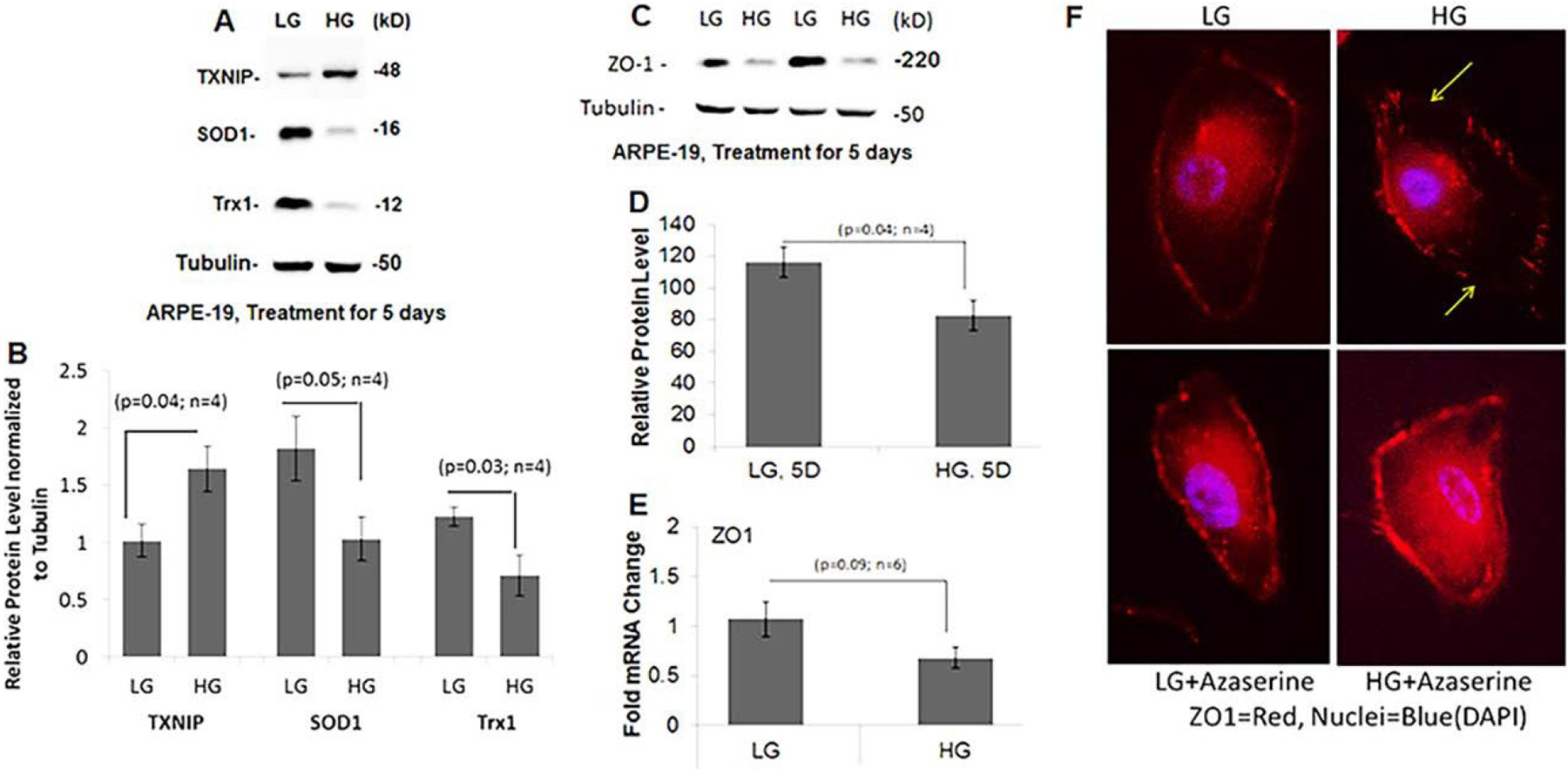
High glucose mediates aberrant redox and tight junction protein expression in APRE-19. ARPE- 19 cells were maintained in DMEM/F-12 medium containing 2% serum with antibiotics containing 5.5mM glucose (LG) or 25mM glucose (HG) for 5 days like those described previously (33). Western blotting and QPCR detected protein and mRNA levels. Data are presented as SEM+/−SE and p value of <0.05 is considered significant when compared between LG and HG using Student’s t-test. (A-B) TXNIP level is increased in HG compared to LG (p<0.04) while that SOD1 and Trx1 are down regulated significantly. Tubulin is used as a control protein. (C-E) Protein and mRNA levels of ZO-1 also decrease in HG compared to LG. (F) Furthermore, ZO-1 immunostaining (red) is also disrupted at the plasma membrane under HG (yellow arrows) when compared to LG. Furthermore, addition of 2μM azaserine (an inhibitor of TXNIP, ref. 25) in the last 24h of the experiment restores ZO-1 plasma membrane staining in HG. A representative of n=3 is shown here.

High glucose alters redox and tight junction protein expression in ARPE-19. We maintained ARPE- 19 cells under LG or HG for 5 days as previously described [33]. As results, on Western blots, we observe that HG significantly increases TXNIP expression, a pro-oxidant protein, when compared to LG while that of the antioxidant enzymes Cu/Zn superoxide dismutase 1 (SOD1) and Trx1 are reduced, indicating cellular redox stress (Figure 1A & 1B). Similarly, the level of tight junction protein, zona occludens 1 (ZO-1), is also reduced under HG both in protein and mRNA levels (Figure 1C–1E). Similar observation is also seen in immunofluorescence staining of ZO-1 in plasma membranes under HG versus LG (Figure 1F, upper panel). Interestingly, azaserine, an inhibitor of the hexosamine biosynthesis pathway and of TXNIP [25], restore ZO-1 plasma membrane staining under HG (Figure 1F, lower panel), indicating that TXNIP may play a role in RPE tight junction leakage and oBRB dysfunction in DR (Figure 1).

**Figure 2:**
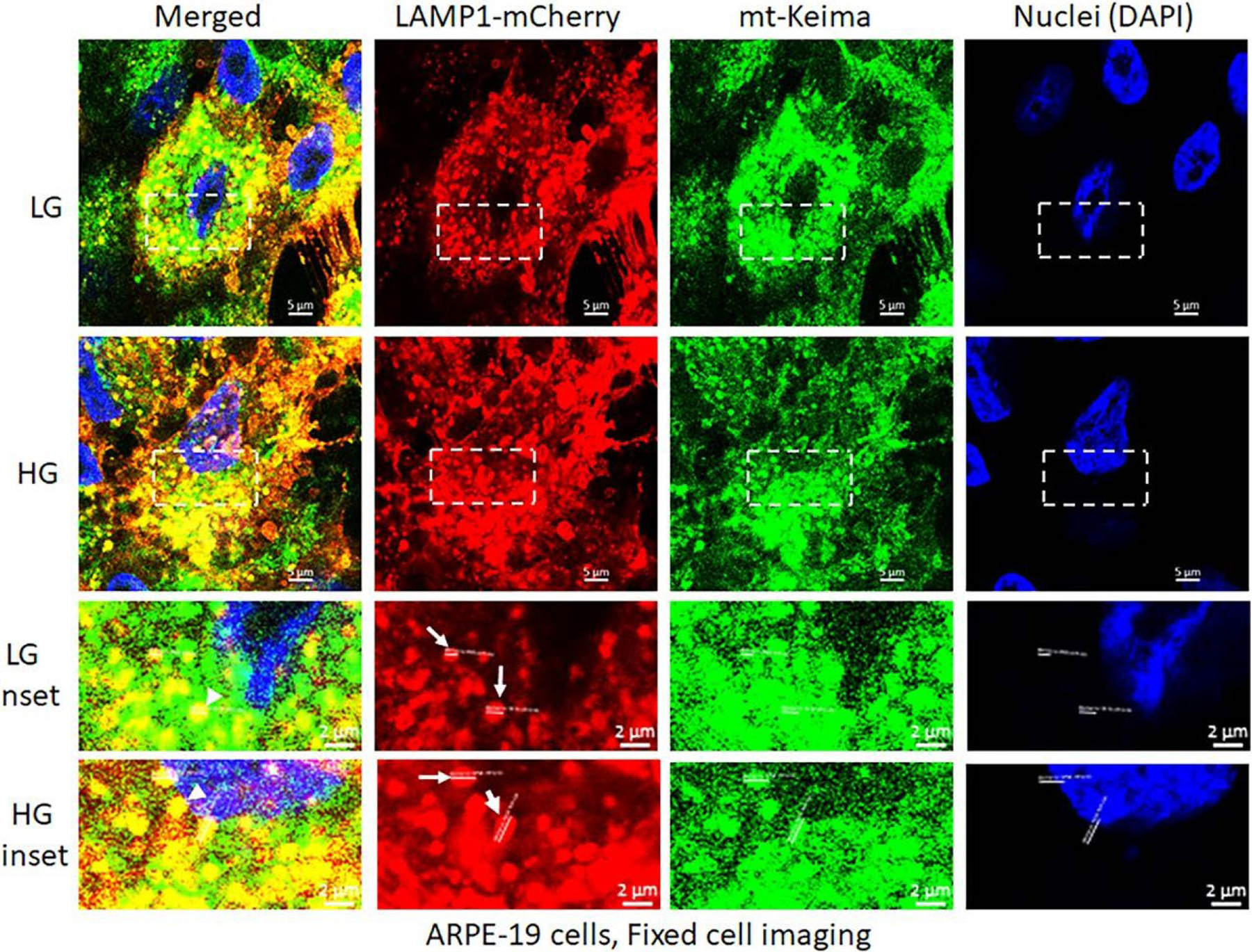
High glucose induces mitophagic flux and lysosome enlargement in ARPE-19 cells. Transduction of mt-Keima and LAMP1-mCherry bearing adenovirus vectors in APRE-19 cells were described previously [33]. Mt-Keima and LAMP1-mCherry vectors were transfected together in ARPE-19 cells and incubated with LG (5.5mM) or HG (25mM glucose) for 5 days, then the cells were fixed, mounted on DAPI containing mounting medium to stain nuclei and imaged with a Zeiss Confocal microscopy at 630x magnification. The images were analyzed by Zen 3.0 blue software and compile in Adobe Photoshop. Under these experimental conditions, mt-Keima emits green both in mitochondria and lysosomes while LAMP1-mCherry emits red in lysosomes. With HG treatment, lysosome sizes are ~1.5- to 2-folds larger than in LG (mCherry, white arrows in insets) while there are also more yellow mito-lysosomes in HG versus LG (green and red combination, arrowheads in insets). A representative of three experiments is shown here.

High glucose induces mitophagic flux and lysosomal enlargement in ARPE-19. We have previously shown that HG causes mitochondrial dysfunction and mitophagic flux in ARPE-19 and primary human HRPE in confocal live cell imaging using the mitochondrial matrix targeted mitophagic probe, mt-Keima [33,34]. Mt-Keima is a coral protein, which emits green light in mitochondrial matrix (neutral or alkaline pH) while it emits red light in acidic lysosomes (pH 4.5–5) after mitophgic flux [35,36]. However, mt-Keima red emission loses if the cells are fixed because the lysosomal acidic environment is disturbed, therefore, emits green. Hence, in this study, we employed co-transduction of adenoviral vectors bearing mt-Keima and LAMP1-mCherry (lysosomal membrane probe, red) in ARPE-19 cells under LG or HG for 5 days. After that, the cells were fixed and imaged in a confocal microscopy for green and red signals. Mt-Keima in fixed cells emits green both in mitochondria and lysosomes while LAMP1-mCherry emits red in lysosomes [33]; therefore, auto/mitolysosomes appear yellow (combination of green and red) while free mitochondria and lysosomes show green and red, respectively. Using this approach, we demonstrate that ARPE-19 under HG, show a greater number of yellow and enlarged autolysosomes than in LG (Figure 2 inset - arrowheads and arrows), suggesting that HG causes mitophagic flux and lysosomal enlargement in RPE cells. This dual labelling of mt-Keima and LAMP1-mCherry may employ in fixed retinal tissues in *in vivo* studies.

**Figure 3:**
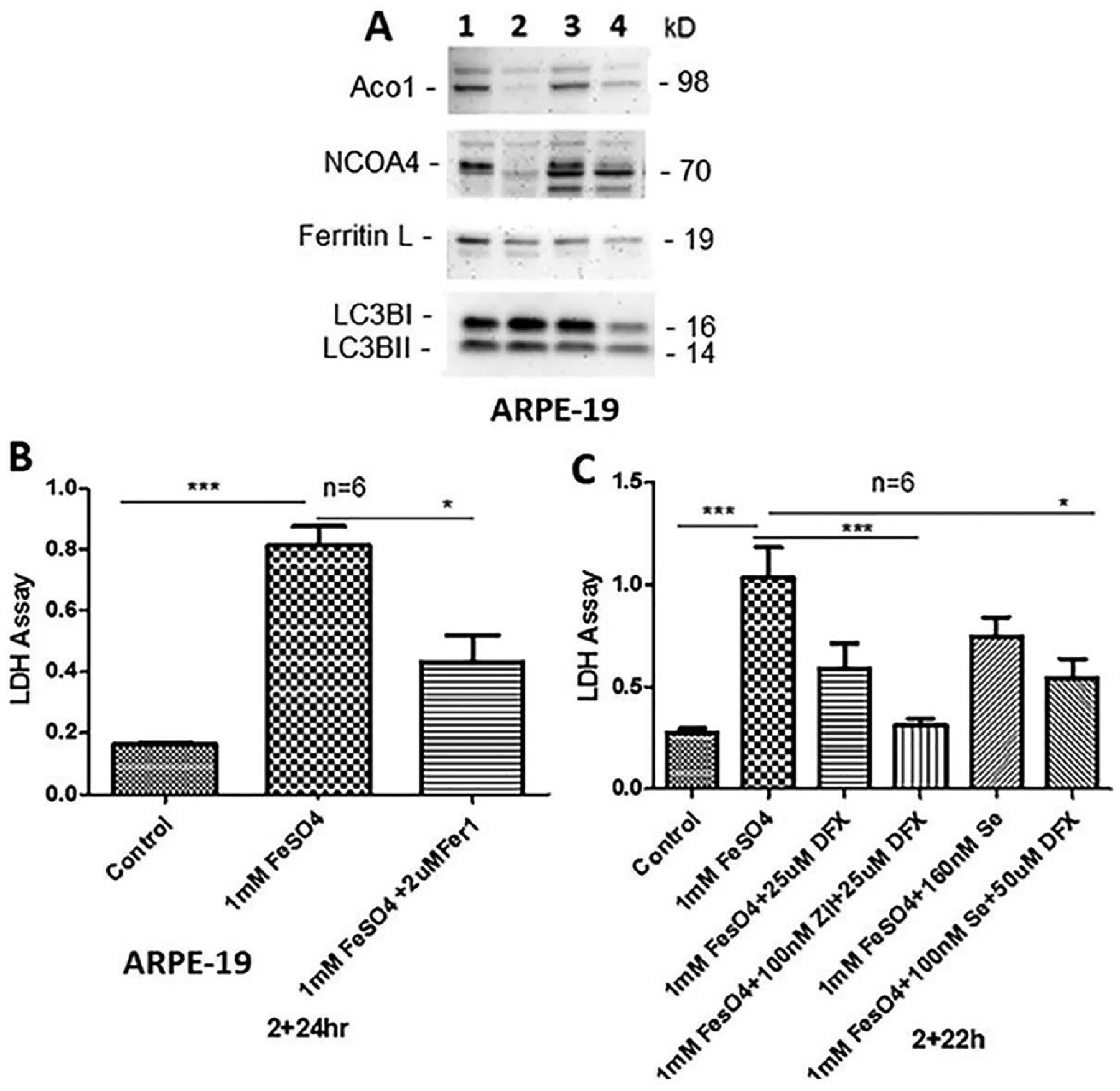
Ferritinophagy and ferroptosis induction by HG and iron-sulfate in ARPE-19. (A) ARPE-19 cells were cultured in LG (1) or HG (2) for 5 days and LG (3) or HG (4) 5 days with 0.5mM hydrogen peroxide (H2O2) in the last 24h of experiment, cells harvested and proteins (30mg) were detected by Western blotting as previously described [33,34]. Cytosolic aconitase (Aco1), ferritinophagy adaptor - nuclear receptor coactivator 4 (NCOA4), and ferritin light chain (Ferritin L) are reduced in HG and H2O2 indicating ferritinophagy induction. HG increases LC3BI, however, H2O2 reduces both LC3BI and II. The blot is a representative n=2. (B) Cell death assay by LDH leakage was performed as described before [34]. Fifty microliters of the medium were assayed for the LDH activity using a commercial LDH assay kit from Pierce (Cat# 88953), according to Manufacturer’s instructions. Incubation of ARPE-19 cells with 1mM FeSO4 increases LDH activity ~5 folds (p<0.05) in media from HG than from LG. Preincubation with ferrostatin 1 (Fer-1, a ferroptosis inhibitor) 2h before adding FeSO4 and present throughout the duration of the experiment, reduces LDH leakage significantly, suggesting ferroptotic cell death in ARPE-19. (C) Similarly, iron chelator deferasirox (25μM DFX), a combination of ALOX-5 inhibitor Zileuton (Zil, 100nM) and DFX (25μM), antioxidant Selenium (Se, 160nM) or a combination of 100nM Se and 50μM DFX reduces LDH activity significantly in ARPE-19 cells, indicating that ferroptosis mechanism may involve various signaling pathways. One-way ANOVA and Bonferroni post hoc test determined differences among means in multiple sets of experiments. Data are presented as SEM+/−SE and p value of <0.05 is considered significant, *<0.01 and ***<0.0001.

Ferritinophagy and ferroptosis occur under HG and iron overloading in ARPE-19. We previously showed that HG-induced TXNIP up-regulation or redox stress by auranofin, which inhibits thioredoxin reductases TrxR1 and TrxR2, in ARPE-19 cells induces mitophagic flux, lysosomal destabilization and NLRP3 inflammasome activation and pyroptosis [34]. Here, we observed that HG causes a reduction in cytosolic aconitase 1 (Aco1), ferritinophagy adaptor NCOA4, and ferritin L levels in ARPE-19, suggesting ferritinophagic flux (Figure 3A). Similar observation is seen with H_2_O_2_ treated ARPE-19, indicating free labile iron may be released under high glucose and oxidative stress environment. To examine if ARPE- 19 undergoes ferroptosis, we treated these cells with 1mM FeSO_4_ for 24h and examine cell death by LDH release in media. The results show that FeSO_4_ causes LDH leakage in ARPE-19, indicating iron-dependent cell death by ferroptosis while pre-incubation (2h before FeSO_4_) ferrostain 1 (Fer-1, 2μM), an inhibitor of ferroptosis, reduces LDH leakage (Figure 3B). In addition, iron chelator deferasirox (DFX, 25μM), Zileuton, ALOX5 inhibitor (Zel, 100nM), and antioxidant Selenium (Se, 160nM) also reduce FeSO_4_-mediated LDH leakage, suggesting several enzymes may participate in this ferroptotic process (Figure 3C). ALOX5 (arachidonate 5-lipoxygenase) is a free iron-containing dioxygenase that catalyzes peroxidation of arachidonic acid, a polyunsaturated fatty acid (PUFA) [22]. Selenium is an antioxidant that is required for the synthesis of selenoproteins (containing Se-cysteine amino acid), such as GPX4, TrxR1 and TrxR2 [37]. Interestingly, Aco1, which is a 4Fe-4S containing enzyme, catalyzes citrate to isocitrate via cis-aconitase in the cytosol like Aco2 in the mitochondrion [38]. However, under oxidative stress, after losing the 4Fe-4S complex, Aco1 acts as an Iron regulatory protein 1 (IRP1), which binds to the 3’-hairpin loops of TfR1 mRNA and stabilizes to increase TfR1 translation and iron uptake [38,39]. The fact that Aco1 level is down by HG in our study may suggest a protective cellular response from free iron increases in the cytosol via mitophagic and ferrritinophagic flux to lysosomes. These results suggest that RPE cells could undergo ferroptotic cell death under chronic hyperglycemia, oxidative stress, and iron overload due to defects in iron metabolic enzymes, transport and ferritinophagy/mitophagy.

### Potential pathways for mitophagy, ferritinophagy and ferroptosis interaction in RPE cells under high glucose environment and oxidative stress:

**Figure 4:**
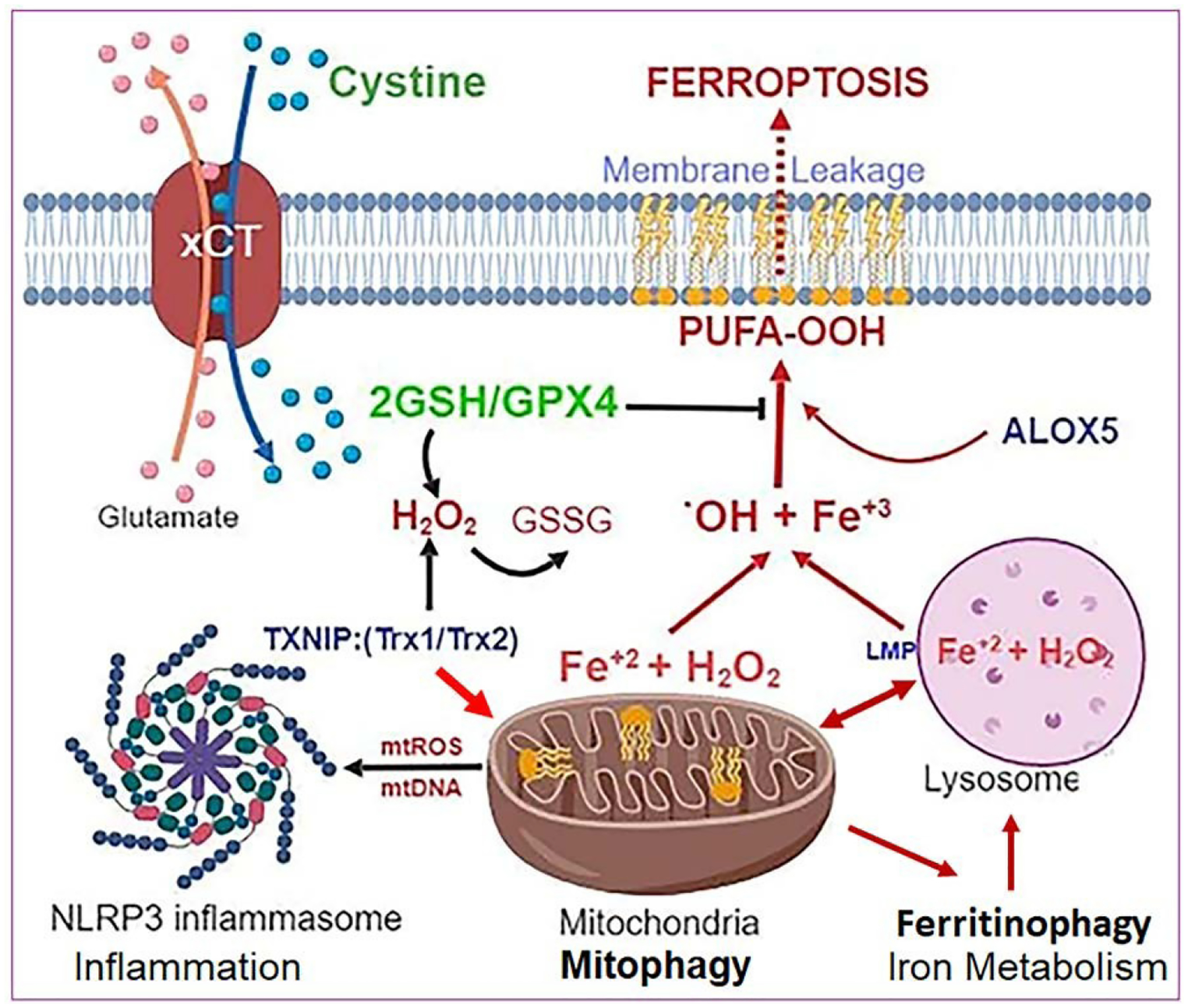
Relationship among Mitophagy, Ferritinophagy and Ferroptosis under oxidative stress. Hyperglycemia-induced TXNIP upregulation and oxidative/nitrosative stress cause mitochondrial damage, ROS generation and mitophagic flux to lysosomes for degradation. A feedback mechanism to replenish mitochondrial mass and bioenergetics may activate ferritinophagy, an autophagic process of degradation and release of free iron Fe^2+^ from ferritin cages in lysosomes for using in the synthesis of mitochondrial Iron-sulfur cluster and heme moeity. However, the released ferrous iron also reacts with H2O2 via Fenton reaction to generate highly reactive hydroxyl radicals (.OH), and biproducts of hydroxide (OH−) and ferric iron (Fe^3+^). These .OH radicals react with cell membranes rich in polyunsaturated fatty acids (PUFA) to mediate lipid peroxidation (PUFA-OOH), including the mitochondria, lysosome and plasma membranes. PUFA-OOH in membranes disturb fluidity and membrane integrity. Free Fe^2+^ may also activate arachidonic 5-lipoxygenase (ALOX5), which generates lipid peroxides (L- OOH). The only enzyme that can remove and prevent lipid peroxidation is GPX4, a selenocysteine protein, using 2 GSH molecules. However, under oxidative stress, GSH level and biosynthesis are down-regulated leading to accumulation of oxidized GSSG. Extracellular cystine (di-cysteine) is transported into cells through plasma membrane glutamate-cystine exchanger (xCT). Down-regulation of xCT, which occurs in DR, will limit cystine import and, therefore, cysteine available for GSH synthesis. Under these conditions, iron-dependent PUFA-OOH due to inhibition of GPX4 activity causes cell death by ferroptosis, a recently identified non-apoptotic cell death. Ferroptosis itself induces an inflammatory response, which assembles the NLRP3 inflammasome, activating caspase-1, IL- 1β/IL-18 and gasdermin-D to cause pyroptotic cell death as well. These events, if unchecked, eventually will sustain a chronic low-grade inflammation, oxidative stress and premature retinal cell death leading to disease initiation and progression of DR and age-related retinal diseases. [Image created with BioRender.com.].

RPE is a single layer of fully differentiated cells that separate the neuroretina from the underlying fenestrated choriocapillaries, forming the oBRB [31,32]. RPE supplies glucose, oxygen and nutrients to photoreceptors while recycling vitamin A (visual pigment). It also phagocytoses photoreceptor outer segment (POS) daily and degrade in lysosomes [40]. Therefore, dysregulation of RPE causes photoreceptor dysfunction and death leading to blindness in various age-related retinal diseases, including DR and AMD [31,32,41]. In this study, we show that (i) high glucose-induced aberrant redox protein expression and oxidative stress in ARPE-19 cause the mitochondrial-lysosomal axis dysregulation and tight junction protein ZO-1 downregulation. (ii) Flux of damaged mitochondria to lysosomes via mitophagy together with activation of ferritinophagy, ferritin autophagy in lysosomes, will result in lysosomal enlargement and subsequent pH increase, lysosomal membrane permeabilization (LMP) and leaking out lysosomal hydrolytic enzymes and undigested materials to the cytosol. One additional event is the release of free labile iron (ferrous Fe^2+^) from lysosomes due to excess mitophagic flux (carrying mitochondrial Fe-S clusters/heme moieties and mtFerritin, mitochondrial iron storage) and from ferritinophagic flux (degradation of ferritin-Fe^3+^ cage in lysosomes). These free Fe^2+^ irons react with H_2_O_2_ to generate highly reactive hydroxyl radicals (·OH) via. Fenton reaction [42]. These highly reactive hydroxyl radicals attack membrane lipids by peroxidation, particularly the polyunsaturated fatty acids (PUFA-OOH), thereby damaging membrane integrity and leaking. The only enzyme that can detoxify phospholipid hydroperoxide to hydroxyl moiety is the redox enzyme GPX4 using 2 GSH molecules. GSH itself is one of the major antioxidants in cells, including mitochondrial matrix, which reduces oxidized proteins and scavenge oxygen radicals. Therefore, TXNIP upregulation in RPE cells under high glucose and downregulation of antioxidant proteins ((SOD1, Trx1) (Figure 1)) will lead to generation of oxidized GSSG and depletion of GSH. Glutathione biosynthesis (a tripeptide of γ-L-glutamyl-L-cysteinyl-glycine) by glutathione synthetase requires cysteine amino acid, which is imported intracellularly as cystine (cysteine-cysteine) via the plasma membrane glutamate- cystine exchanger, xCT, which is down-regulated in DR [43,44]. Along with this, mitochondrial dysfunction, mitophagic flux, ferritinophagy and lysosome destabilization will increase ROS generation and labile iron accumulation within the cell and ferroptosis, which is depicted in Figure 4.

Ferroptosis is a newly defined oxidative cell death mechanism due to iron accumulation and membrane lipid peroxidation due to reduced GSH/GPX4 activity [30]. Ferroptosis itself also is an inflammatory process due to release of cytosolic and nuclear components, such as mitochondrial DNA, single-stranded RNA, mitochondrial heat shock protein HSP60, nuclear HMGB1 and other materials within the cell and extracellularly [45]. Therefore, these cytosolic and nuclear components are recognized by membrane and cytosolic innate immune platforms, such as toll-like receptors (TLR4, TLR7, TLR9, etc.) and inflammasomes, particularly NOD-like NLRP3 and DNA sensing AIM2 [46]. Subsequent activation of caspase-1, IL-1β/IL-18 and gasdermin-D influences oxidative stress and inflammatory cell death by pyroptosis [46,47]. In fact, we demonstrated that auranofin-induced redox stress and mitochondrial dysfunction in ARPE-19 cells cause NLRP3 inflammasome activation and pyroptosis [34]. Moreover, triple combination drug treatment comprising of amlexanox (TBK1/IKKα inhibitor), SS-31 (mitochondria-targeted antioxidant) and tranilast (an inhibitor of NLRP3) reduces redox stress-mediated mitophagic flux, lysosome enlargement and cell death [34]. Therefore, we further propose that targeting TXNIP and the mitochondrial-lysosomal axis dysfunction will also prevent high glucose-induced redox stress, mitochondrial dysfunction, and cell death. To support this hypothesis, we recently found that a triple combination treatment using TXNIP-IN1 (TXNIP-Trx interaction inhibitor), mito-TEMPO (mitochondrial antioxidant) and ML-SA1 (lysosomal transient calcium release channel Mucolipin 1 (MCOLN1/TRPML1), which activates calcineurin phosphatase-lysosomal transcription factor TFEB, prevents high glucose- induced mitochondrial dysfunction, mitophagic flux and lysosome destabilization in a rat retinal Müller cell line, rMC1 (data not presented). TFEB is important for lysosomal biogenesis, autophagic gene expression and mitogenesis via activation of PGC1α expression, which encompasses the Coordinated Lysosomal Expression and Regulation (CLEAR) gene network [48,49].

In conclusion, a slow mitophagy will cause accumulation of damaged mitochondria, which produce less ATP but generate ROS while too fast a mitophagic flux will result in deficiencies in mitochondrial number, bioenergetics and lysosomal overloading and reduced digestive capacity. Therefore, future studies will require identifying an optimal mitophagic flux, which also maintains ferritinophagy and mitochondrial and lysosomal biosynthesis [34,50]. Working out the potential roles of mitophagy, ferritinophagy and lysosome destabilization and organelle communication in retinal cell death by ferroptosis/pyroptosis (including photoreceptor and RPE in DR and age-related retinal diseases) will be helpful in developing combination and organelle targeted drug and gene therapy modalities.

## Figures and Tables

**Table 1:** Reagents and Chemicals.

Name	Company	Cat #
DMEM medium	Mediatech Inc (Manassas, VA)	10-014-CM
F-12 medium	HyClone (Logan, UT)	SH30026.01
Antibiotics and Trypsin	HyClone	
FBS	Corning (Corning, NY)	MT35010CV
Glucose	Sigma (St. Louis, MO)	G8270
Azaserine	Sigma	A-1164
Hydrogen peroxide	Sigma	95321
Mounting medium	Molecular Probes (Eugene, OR)	P36935 FeSO4, Iron (II) sulfate heptahydrate) Sigma 7782-63-0
Ferrostatin 1	Sigma	SML0583
Deferasirox (DFX)	Sigma	SML2673
Zileuton	Sigma	Z4277
Selenium	Sigma	229865

Primary Antibodies	Dilution	Species	Company	Cat # (WB)
TXNIP	1:1000	Rabbit	Cell Signaling (Danvers, MA)	14714S
SOD1	1:1000	Rabbit	Proteintech (Tucson, AR)	10269-1-AP
Trx1	1:500	Rabbit	Santa Cruz	SC-20146
ZO-1	1:1000	Rabbit	Cell Signaling	#13663
Tubulin	1:1000	Rabbit	Cell Signaling	2144S
Aco1	1:1000	Mouse	Proteintech	12406-1- AP
NCOA4	1:1000	Rabbit	Cell Signaling	66849S0
Ferritin L	1:3000	Rabbit	Proteintech	10727-1-AP
LC3B	1:3000	Rabbit	Pierce (Rockville, IL)	L10382

Secondary Antibodies	Dilution	C Company	Cat #
Anti-mouse 2°ry antibody HRP	1:5000 (WB)	S Sigma	A5278
Anti-rabbit 2°ry antibody HRP	1:5000 (WB)	S Sigma	A6154
Anti-goat 2°ry antibody HRP	1:5000 (WB)	S Santa Cruz	sc-2020

IF:					
ZO-1 Primary Ab	1:200	(IF)	Rabbit	Cell Signaling	#13663
Anti-rabbit 2°ry Ab	1:400	(IF)	Goat	Jackson	111-585-176
Alexa Fluor 594				(West Grove, PA)	
